# Anti-inflammatory and antioxidant activities of fractions and compound from *Ricinodendron heudelotii* (Baill.)

**DOI:** 10.1016/j.heliyon.2019.e02779

**Published:** 2019-11-14

**Authors:** Omolara F. Yakubu, Abiodun H. Adebayo, Emeka E.J. Iweala, Isaacson B. Adelani, Temitope A. Ishola, Ying-Jun Zhang

**Affiliations:** a Department of Biochemistry, College of Science and Technology, Covenant University, PMB 1023, Canaan Land, Ota, Ogun State, Nigeria; b State Key Laboratory of Phytochemistry and Plant Resources of West China, Kunming Institute of Botany, Chinese Academy of Sciences, #132, Lanhei Road, Heilongtan, Kunming, China

**Keywords:** Natural product chemistry, Antioxidant, Anti-inflammatory, *Ricinodendron heudelotii*, Corilagin, Tannins

## Abstract

Medicinal plants have been documented over the years to play vital role in promoting human health. The study evaluated the anti-inflammatory and anti-oxidant activities of different fractions and isolated compound from *Ricinodendron heudelotii* leaves. The leaves of *Ricinodendron heudelotii* were extracted with ethanol and further partitioned sequentially using petroleum ether, ethylacetate and butanol. Bioassay–guided fractionation of the ethylacetate fraction was done using repeated column chromatographic technique while the structural elucidation of pure compound was carried out using mass spectra, ^13^C and ^1^H NMR analyses. Antioxidant potential of the fractions and isolated compound were evaluated with 2,2-Azino-bis (3-ethylbenzthiazoline-6-sulfonic acid) (ABTS) and 2,2-diphenyl-1-picrylhydrazyl (DPPH) radical scavenging assays and anti-inflammatory effect of fractions was measured by their inhibitory potency on nitric oxide (NO).

Corilagin, an amorphous tannin was isolated and structurally elucidated. Corilagin showed scavenging effect against ABTS and DPPH radicals which vary in a dose dependent manner. It also showed an antioxidant potential with IC_50_ value of 0.003 mg/mL comparable to vitamin C 0.001 mg/mL) used as standard. The butanol and ethylacetate fractions exhibited significant (p < 0.05) NO inhibition of 60 and 69% respectively after treatment of RAW 264.7 macrophages with lipopolysaccharide. These results demonstrated the role of isolated corilagin as a promising potent antioxidant while the ethylacetate and butanol fractions suppressed the expression of an inflammation mediator by inhibiting nitric oxide.

## Teaser

Natural Product Chemistry, Antioxidant; anti-inflammatory; *Ricinodendron heudelotii*; tannins.

## 1 Introduction

Medicinal plants contain diverse groups of phytochemicals. The therapeutic and physiological effects of these phytochemicals make them suitable for use in treating different diseases [1, 2]. The primary benefit of using medicinal plants and their derivatives is due to their availability and affordability while still offering profound therapeutic benefits. The usage is also based on the belief that herbs might be more effective in treating certain diseases [3]. The therapeutic potential of these plants are linked to one or more compounds they contain which are termed phytochemicals [4].

*Ricinodendron heudelotii* (family: Euphorbiaceae, genus *Ricinodendron*), is commonly called the African oil-nut tree in English, “Okwe” and “Erimado” in Igbo and Yoruba cultures of Nigeria respectively; the Cameroonians call it “Njansang”. Different parts of the tree have been used for treatments with the bark being the most frequently used and the most efficacious. The bark extract is used to cure cough and as antidote to poison [5]. The roots are used as laxative and treatment of stomach pain in Ghana and Nigeria [6]. The bark decoction is used to relieve inflammations and it is equally employed as aphrodisiac. The bark is also used to ease sexual, fertility, menstrual, childbirth pain [6]. The leaves and latex are used to extract guinea worm and as a purgative. It is commonly used in rural areas of Cameroon to prevent abortion. The bark helps cure malaria, yellow fever, and dysentery and equally has diuretic functions [7]. Recently, we reported the antimicrobial effect of *R. heudelotii* extract [8] and the cytotoxic effect of isolated compounds from the leaf extract [9]. It was also reported that the leaf extract exhibited regulatory role on the toxicity of artemisinin when co-administered with artemisinin [10]. Reactive oxygen species (ROS) are generated as a result of oxidative processes in the body. Some of them include hydroxyl and hydrogen peroxide. Oxidative processes are unavoidable as they are important in energy metabolism and utilization of nutrients [11]. Antioxidants help to scavenge free radicals and eliminate them from the body system. Studies have shown a retroverted relationship between disease progression and disease genesis and intake of antioxidant rich foods [12]. Although synthetic antioxidants are available, they are out of reach of many due to high cost, reduced distribution and side effects [13]. Natural antioxidants are however very much available with minimal cost and showing little or no side effects. Compounds high in antioxidant ability are flavonoids and phenols. Trace elements such as copper, manganese and magnesium also act as antioxidants [14]. In small concentrations, ROS are important in the human system as they help in gene expression, regulation of signal transduction and other biological processes [11]. In high concentrations however, ROS can have deleterious effects on biomolecules such as proteins, lipids and other biomolecules and eventually cause cell death [15]. An excess of ROS is also implicated in the genesis of many diseases such as cancer and other age-related diseases including inflammation [15]. Inflammation has been implicated in the genesis or progression of most diseases and medicinal plants have recently been used as potent anti-inflammatory therapy [16]. Inflammation is the body's immune response to foreign substances as well as response to processes such as degeneration and cell death. Inflammation is one of the innate immune responses of the body [17]. Many diseases have been related to the mechanism of oxidative stress and inflammation. It is therefore important to explore the anti-inflammatory and antioxidant abilities of *Ricinodendron heudelotii* which could further add to existing knowledge on the mode of action of the herbal plant.

## 2 Materials and methods

### 2.1 Reagents

2,2^׳^-diphenyl-1-picrylhydrazyl (DPPH), 2,2-azino-bis-3- ethylbenzothiazoline-6-sulfonic acid (ABTS), N^G^ –Methyl-L-arginine acetate salt, 6-hydroxy-2,5,7,8-tetramethylchroman-2-carboxylic acid (Trolox), and ascorbic acid were purchased from Sigma Aldrich (USA). Sephadex LH-20 (25–100 μm, GE Healthcare Bio-Science AB, Uppsala, Sweden), MCI-gel CHP20P (75–150 μm, Mitsubishi Chemical Co, Ltd., Tokyo, Japan), silica gel H-precoated plates, 0.2–0,25 mm thick (Qingdao Haiyang Chemical Co., Qingdao, China).

### 2.2 Plant material

#### 2.2.1 Plant collection

The leaves of *Ricinodendron heudelotii* were sourced within Covenant University environ in Ota, Ogun State, Nigeria. The plant was identified by Dr. J.O. Popoola (a Botanist in Biological Science Department of Covenant University, Ota) and a voucher specimen was prepared and submitted to the Forest Research Institute of Nigeria (FRIN), Ibadan with voucher no FHI 110573. The leaves were allowed to dry at room temperature (25 °C) and blended using an electric blender into coarse powder.

#### 2.2.2 Preparation of plant extract

Extraction of the powdered leaf samples (5 kg) was done via the maceration method using 95% ethanol, the mixture was filtered and the filtrate further condensed under reduced pressure and temperature and the yield of the extract obtained was 11.75%. The concentrated crude extract (0.5 kg) was suspended in 1 L distilled water and partitioned in sequence with petroleum ether (Pet; 8 L), ethylacetate (EtOAc; 8 L) and n-butanol (8 L). The solvent fractions were concentrated to dryness to afford four (4) fractions as: Pet, EtOAc, n-butanol and Water fractions. The EtOAc fraction was selected for subsequent isolation procedures [18].

### 2.3 Isolation of compound from the partitioned fraction

The ethylacetate fraction (246 g) was loaded into a column containing Sephadex LH 20 gel (300 g, 100–200 mesh). The eluent was a mixture of water and methanol in different ratio starting with 100:1. Fractions with similar thin layer chromatography (TLC) patterns were combined to obtain 13 fractions. Fraction 11 (36 g) was chromatographed on a Sephadex LH 20 gel column and then MCI gel by eluting with water:methanol (4:1) to obtain corilagin (12 mg).

### 2.4 Anti-inflammatory activity

Nitric oxide (NO) production from RAW 264.7 macrophages was assessed by measuring accumulation of nitrite in the supernatant after treatment with lipopolysaccharide (LPS) for 24 h with or without the extracts using Griess reagent in a method described by Reif and McCreedy [19]. The experiments were done in triplicates.

#### 2.4.1 Cell culture

RAW 264.7 murine macrophage cells were cultured in Dulbecco's Modified Eagle Media (DMEM) supplemented with 10% foetal bovine serum, streptomycin (100 μg/mL) and penicillin (100 U/mL) in a 5% atmosphere of CO_2_ at 37 °C. Viability of the cells were evaluated via the MTS assay simultaneously.

#### 2.4.2 Procedure

The RAW 264.7 macrophages were seeded into 96 well-microliter plates at a depth of 2 x 10^4^ cells per well. The cells were left overnight to allow them attach properly and then incubated in a medium of LPS (5 μg/mL) (negative control); the compounds were serially diluted up to a maximum concentration of 25 μM in triplicate. After incubation period of 24 h, 100 μL of the supernatant from each well were transferred into new plates containing Griess reagent of equal volume. Absorbance was recorded at 570 nm on a micro plate reader using nitric oxide synthase (NOS) inhibitor, as the positive control (N^G^-Methyl-L-arginine acetate salt (L-NMMA) [19]. Percentage NO inhibition was derived based on each sample's ability to inhibit the production of nitric oxide by RAW 264.7 macrophages when compared with the cells treated with LPS only.

### 2.5 Antioxidant assays

#### 2.5.1 DPPH radical scavenging activity assay

The antioxidant potential of the fractions and isolated compound on 1,1- diphenyl-2-picrylhydrazyl (DPPH) was determined following previously described method by Liu et al. [20]. Methanolic DPPH solution (100 μM, 100 μL) were added to samples of different concentration (1–1000 μg/mL, 100 μL). The mixture was left to stand at room temperature for 15 min and thereafter the absorbance values were read at 517 nm using ascorbic acid as positive control. Six serial dilutions of samples were further prepared and following the above procedure, the absorbance of each sample concentration against a blank was recorded using a microspectrophotometer (Spectra Max 340, California, USA). The radical scavenging activity of DPPH was calculated using the equation below:

DPPH reduction (%) = 100 x [A_blank_ – A_sample_]/A_blank_

Assay was carried out in triplicates and a plot of percentage DPPH reduction and sample concentration was drawn (correlation coefficient R^2^ = 0.99–1). Antioxidant activity was expressed as inhibitory concentration (IC_50_) values.

#### 2.5.2 ABTS radical scavenging activity

The 2.2′-azino-bis (3-ethylbenzothiazoline-6-sulphonic acid (ABTS) radical scavenging activity was accessed following the method described by Re et al. [21]. The working reagent was prepared by dissolving 7 mM ABTS and 2.45 mM potassium persulphate (1:1) in distilled water. The mixture was left in the dark at room temperature for 12–16 h. After which 3 mL of the working reagent was diluted with 150 mL of methanol to obtain an absorbance of 0.700 at 734 nm. The diluted reagent (1 mL) was then added to the sample of varying concentrations (0.2–1.0 mg/mL) and left in the dark to react and the absorbance was measured at 734 nm after 30 min. The assay was done in triplicates using trolox as standard.

Percentage inhibition was calculated as follows:

% ABTS scavenging = ((Abs_control_-Abs_sample_)/Abs_control_) x 100

where Abs_control_ is absorbance of ABTS^+^ + methanol, Abs_sample_ is absorbance of ABTS + methanol.

### 2.6 Statistical analysis

Samples are expressed as mean ± standard error of mean. The values were compared with control group and p values <0.05 were considered significantly different

## 3 Results

### 3.1 Structure elucidation of isolated compound

**Fig. 1:**
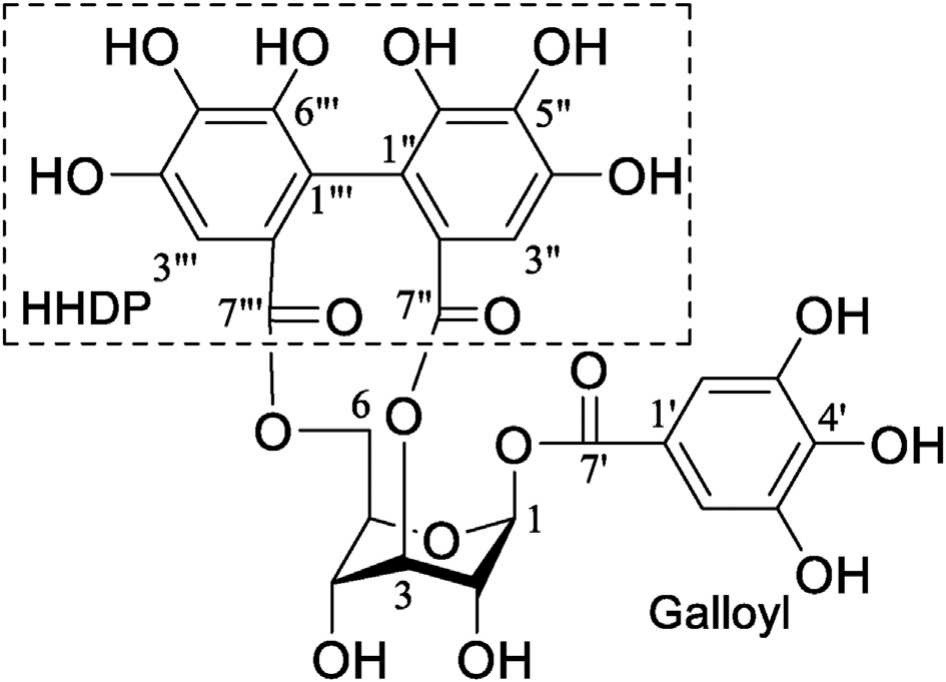
Structure of corilagin isolated from *R. heudelotii*.

The compound was obtained as a yellow amorphous powder, showing a molecular ion peak at *m/z* 633 [M]^−^ in the negative ESI-MS. From ^1^H-, ^13^C-NMR, and DEPT spectroscopic data, the molecular formula of C_27_H_22_O_18_ was deduced. Comparing the data with those in the literature, it was identified as corilagin (Fig. 1) [22].

### 3.2 Spectroscopic data of isolated compound

Corilagin: Yellow amorphous powder, C_27_H_22_O_18_, negative ESI-MS *m/z* 633 [M − H]^−^, ^1^H-NMR (600, CD_3_OD): δ_H_ 7.05 (2H, s, H-2′,6′), 6.69, 6.65 (each 1H, s, HHDP-3″,3‴), 6.36 (1H, d, *J* = 2.4 Hz, H-1), 4.95 (1H, ddd, *J =* 10.9, 8.0, 1.2 Hz, H-6a), 4.80 (1H, brs, H-3), 4.51 (1H, t, *J* = 9.6 Hz, H-5), 4.46 (1H, dd, *J* = 1.7, 1.2 Hz, H-4), 4.16 (1H, dd, *J* = 8.0, 10.9 Hz, H-6b), 3.98 (1H, brd, *J* = 1.2 Hz, H-2); ^13^C-NMR (150 MHz, CD_3_OD): HHDP δ_C_ 167.1 and 167.6 (C=O), 145.8, 145.1 (C-3″, 3‴), 144.9, 144.3 (C-5″,5‴), 136.3, 136.2 (C-4″, 4‴), 116.2, 116.1 (C-1″, 1‴), 124.1, 123.8 (C-2″, 2‴), 107.3, 106.7 (C-6″, 6‴); galloyl δ_C_ 165.2 (C=O), 146.2 (C-3′, C-5′), 139.7 (C-4′), 119.4 (C-1′), 109.7 (C-2′, C-6’); glucose δ_C_ 92.9 (C- 1), 78.3 (C-3), 76.6 (C-5), 72.4 (C-2), 64.2 (C-6), 62.8 (C-4).

### 3.3 Inhibition of nitric oxide production by *R. heudelotii*

The effects of the fractions of *R. heudelotii extract* on the nitric oxide production in the macrophage cells were evaluated 24 h after cells were treated with 1 μg/mL of LPS and 40 μg/mL of the fractions of *R. heudelotii*. As reported in Table 1, the ethylacetate fraction significantly decreased the production of nitric oxide. We can infer from this study that *R. heudelotii* can inhibit production of nitric oxide in RAW 264.7 macrophages when stimulated with LPS.

**Table 1:** Percentage Inhibition of extract of *R. heudelotii* on Nitric oxide production.

	Conc %	Inhibition
L-NAME	50 μM	31.11 ± 0.40
Crude extract	40 μg/mL	52.45 ± 1.07
Ethylacetate fraction	40 μg/mL	69.03 ± 1.54*
Water fraction	40 μg/mL	30.29 ± 0.91
Butanol Fraction	40 μg/mL	60.66 ± 1.25*

* L-NAME: N omega-nitro-L-arginine methyl ester.

*significant (p < 0.05) when compared with the control.

% inhibition represented as mean ± SEM of 3 replicates.

### 3.4 *In vitro* antioxidant assessment of isolated compound and fractions of *R. heudelotii*

**Fig. 2:**
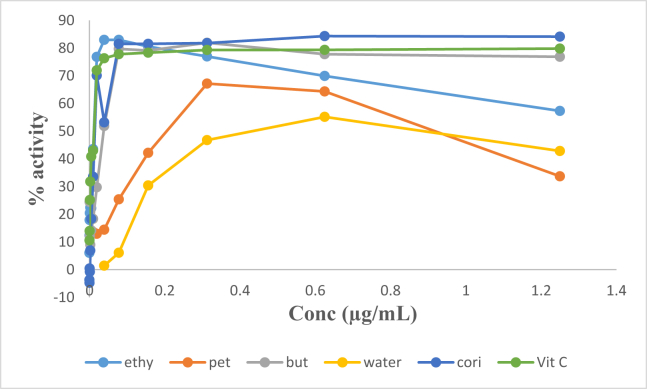
IC_50_ values of DPPH radical scavenging activity of extracts and compound from *R. heudelotii*.

The *in vitro* antioxidant activity of the butanol and ethylacetate fractions were highly comparable with vitamin C (Fig. 2).

**Fig. 3:**
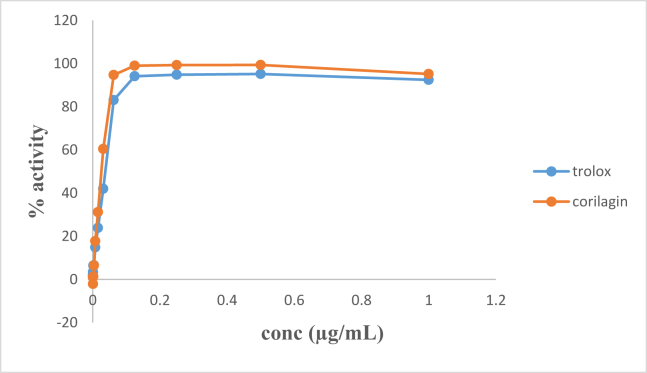
IC_50_ values of ABTS activities of corilagin from *R. heudelotii*.

The antioxidant activity of corilagin isolated from *R. heudelotii* was slightly higher than that of the standard used; vitamin C for DPPH (Fig. 3a) and trolox for ABTS (Fig. 3b) assay. The result also showed that it is concentration dependent (Fig 3a and b). In both methods, corilagin showed potential antioxidant activity.

## 4 Discussion

Free radicals like H_2_O_2_ and NO produced during inflammation can result in cell damage and vasodilation which can lead to chronic inflammation; thus placing much importance on a potent anti-inflammatory agent. This study evaluated the anti-inflammatory effect of different fractions of *R. heudelotii* in activated RAW 264.7 macrophages as well as antioxidant activity of the fractions and isolated compound, corilagin on DPPH and ABTS dyes.

Nitric oxide is considered to mediate inflammatory reactions due to it’s over production in diseased conditions [23] and can contribute to chronic inflammatory disease of the gut, joints and lungs. Hence NO inhibitors could serve as a therapeutic measure in managing inflammatory diseases [23, 24].

In this current study, the ethylacetate and butanol fractions exhibited significant (p < 0.05) NO inhibition after treating the RAW 264.7 macrophages with LPS. The ethylacetate fraction showed the highest percentage inhibition of 69 while the butanol fraction exhibited 60% inhibition when compared with the control (N^G^-Methyl-L-arginine acetate salt) (Table 1). The NO inhibitory effect of the ethylacetate fraction may help arrest series of reactions originated as a result of excess peroxynitrite generation which is deleterious to health, thus can be employed as a potential ameliorative agent for inflammation [14].

The qualitative phytochemical screening of the butanol and ethylacetate fractions of *R. heudelotii* revealed the presence of tannins, flavonoids amongst others as reported by Yakubu et al. [8]. This might account for the antiinflammatory potential of these two fractions. This correlates with the findings of Ahmadiani et al. [25] which showed that tannins and flavonoids exhibit anti-inflammatory effects. The water fraction was less effective in NO inhibition and this could be due to absence of tannins.

From our study, the isolated corilagin was obtained from the ethylacetate fraction of *R. heudelotii*. The presence of corilagin, a tannin could have played a crucial role in the NO inhibitory effect as seen in Table 1 [26]. Corilagin has also been shown to lower pro-inflammatory cytokines and mediators such as iNOS and TNF-α by blocking the NF-kb pathway in macrophage cell lines stimulated by lipopolysaccharide (LPS) [27, 28]. It also reduced the effect of N9 murine cells injury induced by tert-butyl hydroperoxide (TBHP) [29].

In addition, we observed that the butanol and ethylacetate fraction including the isolated compound had highly comparable antioxidant effect on two different oxidant dyes (ABTS and DPPH) when compared with trolox and ascorbic acid which were standards for ABTS and DPPH respectively (Figs. 2 and 3). Measurement of the antioxidant activity via DPPH and/or superoxide scavenging activities has been reported to have a linear correlation with such plant's phenolic content [30, 31]. This implies that the high antioxidant activity displayed by the ethylacetate and butanol fractions could be as a result of phenolic components present in each of the fractions. Also the possibility of multiple phenolic compounds in the ethylacetate fraction working together to scavenge DPPH cannot be ignored. The antioxidant activity of corilagin isolated from *R. heudelotii*; a phenolic tannin, was observed to be slightly higher than Vitamin C, the standard control used for this study; the result also showed that it is concentration dependent (Fig. 3). The high activities of the compound could be linked to the high level of reactivity of its hydroxyl group as Jayant et al. [32] reported that antioxidant activity can increase with increased number of hydroxyl groups. This is in accordance with the study carried out by Chen and Chen [29] where they suggested corilagin as a candidate for treating neurodegenerative diseases by attenuating oxidative stress injury induced by tert-butyl hydroperoxide in microglial cells. The anti-tumor potential of corilagin has also been reported by Hau et al*.* [33] *in vivo* in hepatocellular carcinoma (Hep3B) due to its antioxidant potential.

## 5 Conclusion

As seen in Fig. 1, corilagin has a large number of hydroxyl groups; hence we can conclude that our results demonstrated the role of isolated corilagin as a promising potent antioxidant while the ethylacetate fraction and butanol fractions suppressed the expression of an inflammation mediator by inhibiting nitric oxide. Further studies might be conducted to explore the possible mode of action of corilagin in interacting with the oxidative and inflammatory pathways in animal and cell models.

## Declarations

### Author contribution statement

Abiodun Humphrey Adebayo, Emeka E. J. Iweala: Conceived and designed the experiments; Wrote the paper.

Omolara Faith Yakubu: Performed the experiments; Analyzed and interpreted the data; Wrote the paper.

Isaacson B. Adelani: Analyzed and interpreted the data.

Temitope A. Ishola: Contributed reagents, materials, analysis tools or data; Wrote the paper.

Ying-Jun Zhang: Conceived and designed the experiments; Contributed reagents, materials, analysis tools or data.

### Funding statement

This research did not receive any specific grant from funding agencies in the public, commercial, or not-for-profit sectors.

### Competing interest statement

The authors declare no conflict of interest.

### Additional information

No additional information is available for this paper.
